# Epidemiological trends of elbow and forearm injuries in high school baseball and softball players

**DOI:** 10.1016/j.xrrt.2025.04.006

**Published:** 2025-05-08

**Authors:** Christopher Frey, John Kriz, Aaron Sciascia, Candler Mathews, Eric Bowman

**Affiliations:** a Department of Orthopaedic Surgery, Vanderbilt University Medical Center, Nashville, TN, USA; b Vanderbilt University, Nashville, TN, USA; c Institute for Clinical Outcomes and Research, Lexington Clinic, Lexington, KY, USA

**Keywords:** Baseball, Softball, Elbow, Forearm, High school, Epidemiology, Injury prevention

## Abstract

**Background:**

Elbow and forearm injuries are common in high school baseball and softball players. Given the trends in overuse and early sport specialization, it may be prudent to provide an updated review of the epidemiological data to aid injury prevention efforts.

**Methods:**

Data regarding elbow and forearm injuries in high school baseball and softball players from the 2005-2006 through 2018-2019 seasons was extracted from the National High School Sports-Related Injury Surveillance Study.

**Results:**

There were 518 total elbow and forearm injuries reported for 5,738,470 athletic exposures (AEs) yielding an overall injury rate of 0.90/10,000 AEs. Baseball had an increase of 0.044 elbow/forearm injury increase per 10,000 AEs per year (*P* = .04, 95% Confidence Interval: 0.03, 0.085) while softball did not have a significant change. Baseball had a significantly higher injury rate in competition than practice (Injury rate ratio = 2.29, 95% CI: 1.86, 2.83). While there was no significant difference in overall injury rate for baseball pitchers than nonpitchers, softball nonpitchers had a significantly greater injury rate than pitchers (Injury rate ratio = 0.25, 95%; CI: 0.15, 0.42).

**Conclusion:**

This study found that overall forearm and elbow rate of injury has increased for baseball since the 2005-2006 season. There was no significant trend in softball. Softball nonpitchers had a higher rate of injury than pitchers, while there was no difference between positions in baseball. This trend and difference between sports and positions imply the importance of injury prevention efforts tailored to each athlete.

Baseball remains a popular sport among youth athletes in the United States, with an estimated nearly 3.3 million participants (11.5%) in 2022.^13^ High school (HS) softball and baseball players are at risk for varied injuries about the elbow, given their range in skeletal maturity.^11^ Elbow pathologies such as medial ulnar collateral ligament (UCL) injuries, apophysitis, and osteochondritis dissecans can all occur in this age group. In addition to the time away from sport and direct morbidity of the injury and treatment, there are psychological costs to injuries in the young athlete.^16^ Thus, elbow injuries in HS baseball and softball players may represent a considerable public health issue affecting the mental and physical well-being of young athletes.

At the collegiate and HS levels, UCL injuries are presumed to be on the rise as well, as the workload on individual athletes has increased in recent years.^14^ Collegiate programs have seen dramatic impacts, with 56.8% of programs losing at least 1 player per year to UCL injury. At the adolescent and HS level, various risk factors including skeletal age, biomechanics, and time of year can dictate how susceptible an athlete is to a throwing injury. However, an increased workload due to more tournaments, games, practice hours, strength and conditioning, and throwing programs has changed the playing field of HS throwing athletes.

Considering this, there is a clear need to identify trends and risk factors of these injuries so researchers, trainers, and athletes can more accurately make adjustments in training and workload to prevent injury.^3^ As of the time of writing, there have been no epidemiological studies analyzing HS baseball and softball elbow and forearm injury rates in the United States since the 2014-2015 season. Some studies have analyzed fatigue, specialization, showcase participation, and high pitch velocities as additional risk factors, but much of this is based upon data from a decade ago.^15^ Analysis of more recent trends is needed to update understanding and prevention of elbow injuries in HS throwing athletes.

The objective of this study is to describe and analyze trends in elbow injuries in HS softball and baseball athletes. We hypothesize that there will be an overall increase in the rate of injuries for both baseball and softball players.

## Methods

### Data collection

Institutional review board approval was obtained before beginning the study. Data were obtained from the National High School Sports-Related Injury Surveillance Study using HS Reporting Information Online database. This is an internet-based surveillance system where certified athletic trainers (AT) report sports-related injuries and athlete-exposures.^8^ It has previously been used for baseball amongst other sports.^10^ Data were available and collected for the years 2005-2019.

### Definitions

Athletic exposures (AE) were defined as an event where an athlete participates in a school-sanctioned practice or competition, regardless of amount of play in that particular event. AE data was provided in total by year, competition, and practice. Player position was reduced from individual specific positions to pitchers and nonpitchers (includes all field positions including designations of batter, baserunner, and non-field player ie player sitting in dugout at time of injury). Injuries were designated contact injuries if they were from contact with another player, a ball, or a base. Medical disqualification included disqualification for season, career, athlete chose to not continue, and athlete released from the team. If the season ended before the athlete returned, a variable was available asking for the clinician's opinion as to which outcome would likely have occurred if the season did not ended that is 1-6 days. This variable was imputed when available for any missing outcome. Diagnosis variables were combined as follows:

• Contusion = contusion
• Dislocation = dislocation, subluxation
• Fracture = fracture, stress fracture, apophysitis, avulsion
• Inflammation = inflammation, epicondylitis, tendonitis, bursitis
• Nerve = nerve
• Other = ruptured bursa, osteochondritis dissecans, bone spur, overuse, multiple injuries
• Sprain = sprain, sprain partial, sprain complete, hyperextension
• Strain = strain, strain muscle, strain tendon, strain partial muscle, strain partial tendon
• Wound = laceration, skin infection

### Statistical analysis

Descriptive statistics were calculated for demographic variables, reporting means and standard deviations (SDs) for continuous variables whereas counts and frequencies were reported for categorical variables. In the event that age, height, or weight was not reported, mean imputation procedures were employed. First, the mean and SD was calculated for each variable by sport. Next, the mean, upper value, and lower value of the SD was coded as 1, 2, and 3, respectively. Finally, a random number generator was used to identify a coded value to be imputed for each missing data point. Appropriate univariate analyses were performed comparing baseball to softball players.

For determining incidence and its trend over time, a linear-by-linear trend analysis was performed to determine if the injury rates for baseball and softball increased or decreased annually. Trends were calculated in relation to all annual exposures combined, as well as by exposures from competition and practice individually. Injury rates and 95% confidence intervals (95% Confidence Interval) were calculated for using the formula: (Number of injuries/AE) × 1000. Injury rates were calculated for total injuries and for each sport individually. In addition, injury rates were calculated by time missed and by diagnosis for baseball, softball, and both sports combined. Incidence rate ratios (IRRs) with 95% CIs comparing competition to practice and baseball to softball were calculated within and between sports.

In assessing relative risk, the same IRR calculations noted earlier were performed for pitchers and nonpitchers. A multivariate logistic regression was employed to evaluate if player position (pitcher or nonpitcher), event type (competition or practice), age, height, weight, and sport (baseball or softball) were associated with the occurrence of elbow injury. Multicollinearity was assessed via variance inflation testing. If any variable exceeded 2.5, then it would be removed from the model. After model construction, the Hosmer–Lemeshow goodness-of-fit test was performed as it is specific to logistic models and determines whether the underlying covariate patterns are above and beyond what is expected given the data. If the goodness-of-fit was not statistically significant, then the model was retained. Logistic regression results are presented as odds ratios and 95% CIs. All analyses were performed with STATA 18.5 SE (STATACorp, Inc., College Station, TX). Statistical significance was set a priori as *P* ≤ .05.

## Results

**Figure 1:**
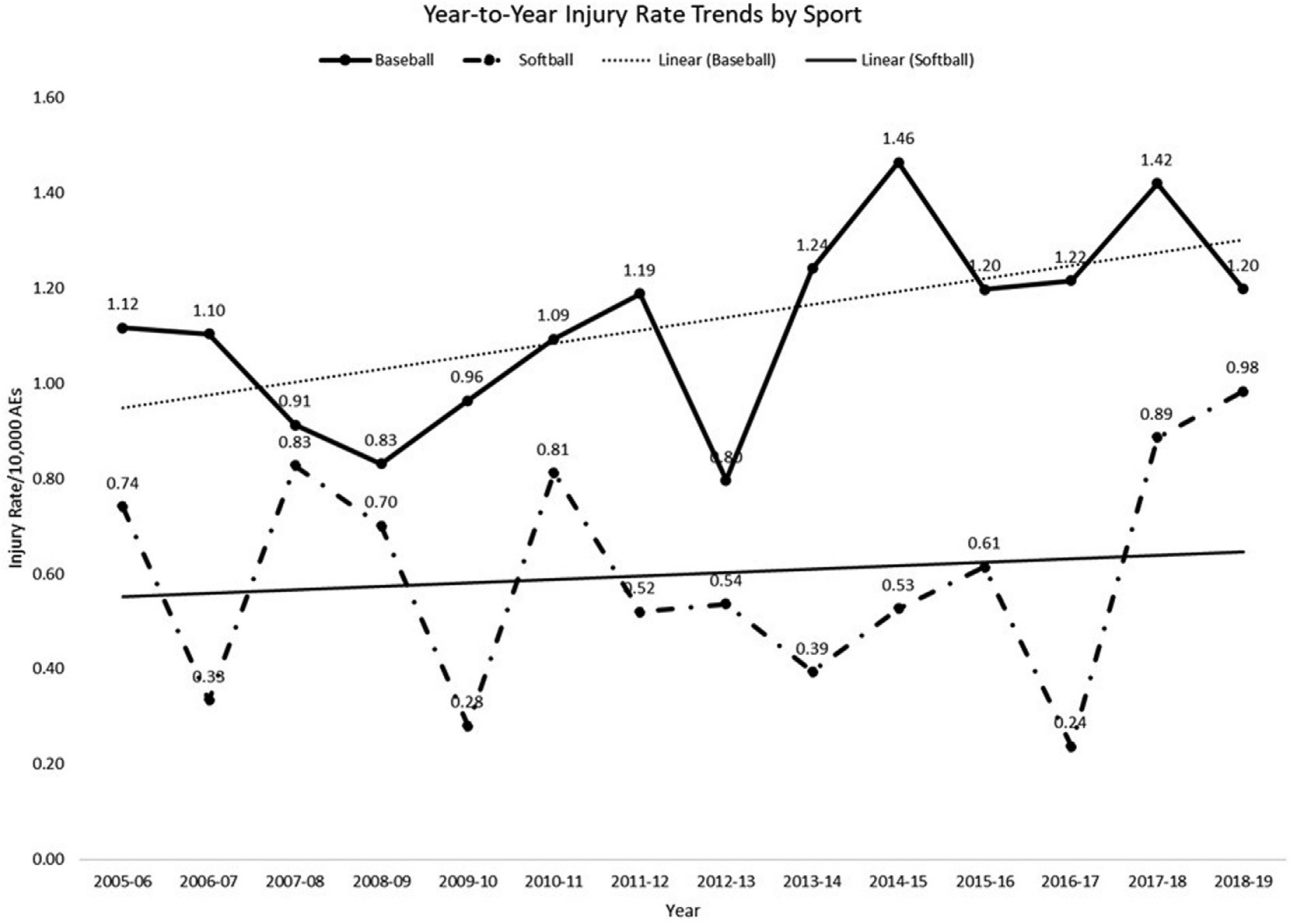
Baseball and softball injury rate trend over time.

There were 518 total injuries reported between 2005-2019 within the dataset with 419 occurring to the elbow and 99 occurring to the forearm (Table I). There was a statistically significant linear trend in baseball with an average yearly increase of 0.044 elbow/forearm injury increase per 10,000 AEs when accounting for all exposures (*P* = .04, 95% CI: 0.030, 0.085) (Fig. 1). This trend was not significant for competition or practice injuries in isolation. There was no significant change in softball injuries per exposure over the same time period.

**Table I:** Demographic information for entire cohort.

Descriptor	Overall n = 518	Baseball n = 372	Softball n = 146	*P* value
Age (yr)				
Mean ± SD	16.0 ± 1.2	16.1 ± 1.2	15.8 ± 1.2	.01
Missing	6	5	1	
Height (cm)				
Mean ± SD	174.2 ± 8.6	177.0 ± 7.4	167.1 ± 6.4	<.001
Weight (kg)				
Mean ± SD	71.9 ± 12.1	75.3 ± 11.4	63.5 ± 9.6	<.001
School Grade				
8^th^	3 (0.6%)	1 (33.3%)	2 (66.7%)	.14
9^th^	123 (24.2%)	79 (64.2%)	44 (36.8%)	.04
10^th^	141 (27.8%)	97 (68.8%)	44 (31.2%)	.38
11^th^	119 (23.4%)	91 (76.5%)	28 (23.5%)	.18
12^th^	122 (24.0%)	96 (78.7%)	26 (21.3%)	.05
Missing	10	8	2	---
Position				
Baserunner	10 (1.9%)	7 (70.0%)	3 (30.0%)	.90
Batter	55 (10.6%)	40 (72.7%)	15 (27.3%)	.87
Pitcher	198 (38.2%)	165 (83.3%)	33 (16.7%)	<.001
Catcher	58 (11.2%)	35 (60.3%)	23 (39.7%)	.04
First	24 (4.6%)	14 (58.3%)	10 (41.7%)	.13
Second	26 (5.0%)	18 (69.2%)	8 (30.8%)	.76
Shortstop	18 (3.5%)	15 (83.3%)	3 (16.7%)	.27
Third	21 (4.1%)	15 (71.4%)	6 (28.6%)	.97
Leftfield	33 (6.4%)	20 (60.6%)	13 (39.4%)	.14
Center	25 (4.8%)	17 (68.0%)	8 (32.0%)	.66
Rightfield	17 (3.3%)	7 (41.2%)	10 (58.8%)	.004
Nonfield/other	8 (1.6%)	5 (62.5%)	3 (37.5%)	.56
Unknown/missing	25	14	11	---
MOI				
Contact	162 (31.4%)	108 (66.7%)	54 (33.3%)	.07
Illness	2 (0.4%)	1 (50.0%)	1 (50.0%)	.49
Noncontact	136 (26.4%)	114 (83.8%)	22 (16.2%)	<.001
Other	9 (1.7%)	8 (88.9%)	1 (11.1%)	.25
Overuse/chronic	207 (40.1%)	140 (67.6%)	67 (32.4%)	.08
Missing	2	1	1	---

*SD*, standard deviation; *MOI*, mechanism of injury.

### Injury rates by sport

The total injuries, total AEs, and injury rates for all injuries are detailed in Table II. The total injury rate for both sports combined was 0.90/10,000 AEs (95% CI = 0.83, 0.99). The injury rates were significantly greater for competition compared to practice for both sports combined (IRR = 1.94, 95% CI: 1.63, 2.31). The injury rates for baseball alone were also significantly greater for competition than practice (IRR = 2.29, 95% CI: 1.86, 2.83), but not for softball. Baseball was noted to have significantly greater rates of injury than softball for all injuries (IRR = 1.90, 95% CI: 1.56, 2.31).

**Table II:** Total and sport-specific elbow/forearm injury counts and rates.

Event	Injuries	Exposures	Rate/10000 Exposures (95% CI)	IRR
Overall				
Competition	264	2,001,975	1.32 (1.16, 1.48)	**1.94 (1.63, 2.31)**
Practice	256	3,736,495	0.68 (0.60, 0.76)	
Total	518	5,738,470	0.90 (0.82, 0.98)	
Baseball				
Competition	206∗	1,155,822	1.78 (1.543, 2.03)	**2.29 (1.86, 2.83)**
Practice	166†	2,134,006	0.78 (0.66, 0.90)	
Total	372‡	3,289,828	1.13 (1.02, 1.25)	
Softball				
Competition	58	846,153	0.69 (0.51, 0.86)	1.25 (0.88, 1.76)
Practice	88	1,602,489	0.55 (0.43, 0.66)	
Total	146	2,448,642	0.60 (0.50, 0.69)	

*IRR*, incidence rate ratio (compares competition to practice); *CI*, confidence interval.

Bolded values indicate statistical significance.

∗ IRR 2.60 (95% CI:1.93, 3.54) significantly greater for baseball compared to softball *P* < .001.

† IRR 1.41 (95% CI:1.09, 1.86) significantly greater for baseball compared to softball *P* < .001.

‡ IRR 1.90 (95% CI:1.56, 2.31) significantly greater for baseball compared to softball *P* = .008.

### Injury rates by time missed and diagnosis

The greatest number of injuries resulted in 1-6 days of missed time from sport (combined and per each sport) with an overall injury rate of 0.37/10,000 AEs (95% CI: 0.32, 0.42) (Table III). Compared to softball, baseball had significantly greater rates for injuries resulting in 10-21 days missed (IRR = 3.58, 95% CI: 2.07, 6.58), 22 or more days missed (IRR = 4.54, 95% CI: 2.31, 9.94), and medical disqualification (IRR = 5.71, 95% CI: 2.43, 16.35).

**Table III:** Total and sport-specific injury rates by time missed.

Time Missed (d)	Overall			Baseball			Softball			IRR
	Injuries	Exposures	Rate/10000 Exposures (95% CI)	Injuries	Exposures	Rate/10000 Exposures (95% CI)	Injuries	Exposures	Rate/10000 Exposures (95% CI)	
1-6	213	5,738,470	0.37 (0.32, 0.42)	123	3,289,828	0.37 (0.31, 0.44)	89	2,448,642	0.36 (0.29, 0.44)	1.03 (0.78, 1.37)
7-9	78	5,738,470	0.14 (0.11, 0.17)	53	3,289,828	0.16 (0.12, 0.20)	24	2,448,642	0.10 (0.06, 0.14)	1.64 (1.00, 2.78)
10-21	93	5,738,470	0.16 (0.13, 0.20)	77	3,289,828	0.23 (0.18, 0.29)	16	2,448,642	0.07 (0.03, 0.10)	**3.58 (2.07, 6.58)**
22 or more	71	5,738,470	0.12 (0.09, 0.15)	61	3,289,828	0.19 (0.14, 0.23)	10	2,448,642	0.04 (0.02, 0.07)	**4.54 (2.31, 9.94)**
MDQ	52	5,738,470	0.09 (0.07, 0.12)	46	3,289,828	0.14 (0.10, 0.18)	6	2,448,642	0.02 (0.01, 0.04)	**5.71 (2.43, 16.35)**

*IRR*, incidence rate ratio (compares baseball to softball); *CI*, confidence interval; *MDQ*, medical disqualification/left team.

Bolded values indicate statistical significance.

Injury rates by body part and diagnosis are located in Table IV. Overall, sprains (134), inflammatory conditions (104), contusions (99), and strains (87) were the most common diagnoses for both sports combined and individually.

**Table IV:** Total and sport-specific elbow/forearm injury counts and rates by diagnosis.

Diagnosis	Overall			Baseball			Softball			IRR
	Injuries	Exposures	Rate/10000 Exposures (95% CI)	Injuries	Exposures	Rate/10000 Exposures (95% CI)	Injuries	Exposures	Rate/10000 Exposures (95% CI)	
Contusion	99	5,738,470	0.17 (0.14, 0.21)	59	3,289,828	0.18 (0.13, 0.23)	40	2,448,642	0.16 (0.11, 0.21)	1.10 (0.72, 1.68)
Dislocation/Subluxation	3	5,738,470	0.01 (−0.00, 0.01)	2	3,289,828	0.01 (−0.00, 0.01)	1	2,448,642	0.01 (−0.00, 0.01)	1.49 (0.07, 87.82)
Fracture	51	5,738,470	0.09 (0.06, 0.11)	42	3,289,828	0.13 (0.09, 0.17)	9	2,448,642	0.04 (0.01, 0.06)	**3.47 (1.67, 8.12)**
Inflammation	104	5,738,470	0.18 (0.15, 0.22)	64	3,289,828	0.19 (0.15, 0.24)	40	2,448,642	0.16 (0.11, 0.21)	1.19 (0.79, 1.81)
Nerve	16	5,738,470	0.03 (0.01, 0.04)	12	3,289,828	0.04 (0.02, 0.06)	4	2,448,642	0.02 (0.0, 0.03)	2.23 (0.68, 9.50)
Other	15	5,738,470	0.03 (0.01, 0.04)	13	3,289,828	0.04 (0.02, 0.06)	2	2,448,642	0.01 (−0.00, 0.02)	**4.84 (1.10, 44.16)**
Sprain	134	5,738,470	0.23 (0.19, 0.27)	109	3,289,828	0.33 (0.27, 0.39)	25	2,448,642	0.10 (0.06, 0.14)	**3.25 (2.09, 5.23)**
Strain	87	5,738,470	0.15 (0.12, 0.18)	63	3,289,828	0.19 (0.14, 0.24)	24	2,448,642	0.10 (0.06, 0.14)	**1.95 (1.20, 3.27)**
Wound	9	5,738,470	0.02 (0.01, 0.03)	8	3,289,828	0.02 (0.01, 0.04)	1	2,448,642	0.01 (−0.00, 0.01)	5.95 (0.80, 264.22)

*IRR*, incidence rate ratio (compares baseball to softball); *CI*, confidence interval.

Bolded values indicate statistical significance.

Fracture = traditional fracture, stress fracture, apophysitis, avulsion; Inflammation = bursitis, inflammation, tendinitis; Other = ruptured bursa, osteochondritis dissecans, bone spur, overuse, multiple injuries; Wound = laceration, skin infection.

### Injury rates by position

The total injuries, total AEs, and injury rates for all injuries that occurred by pitchers and nonpitchers are detailed in Table V. The total injury rate for both sports combined was 0.30/10,000 AEs (95% CI = 0.25, 0.34) for pitchers and 0.40/10,000 AEs (95% CI = 0.35, 0.45) for nonpitchers. There were significantly greater injury rates that occurred overall (IRR = 0.75, 95% CI: 0.61, 0.92) and during practice (IRR = 0.53, 95% CI: 0.39, 0.72) for nonpitchers. There were no significant differences that occurred in injury rates between pitchers and nonpitchers for baseball. However, softball nonpitchers had significantly greater injury rates for all injuries (IRR = 0.25, 95% CI: 0.15, 0.42) during competition (IRR = 0.44, 95% CI: 0.20, 0.93), and during practice (IRR = 0.17, 95% CI: 0.07, 0.34) compared to softball pitchers. The results of the multivariate logistic regression revealed elbow injuries were associated with being a pitcher (odds ratio = 1.75, 95% CI: 1.05, 2.93, *P* = .03) and participating in practice (odds ratio = 2.07, 95%CI: 1.27, 3.36, *P* = .003), while controlling for age, height, weight, and sex (Table VI).

**Table V:** Total and sport-specific elbow/forearm injury counts and rates by position.

Event	Pitchers			Nonpitchers		
	Injuries	Exposures	Rate/10000 Exposures (95% CI)	Injuries	Rate/10000 Exposures (95% CI)	IRR∗
Overall						
Competition	101	2,001,975	0.50 (0.41, 0.60)	97	0.48 (0.39, 0.58)	1.04 (0.78, 1.39)
Practice	70	3,736,495	0.19 (0.14, 0.23)	131	0.35 (0.29, 0.41)	**0.53 (0.39, 0.72)**
Total	171	5,738,470	0.30 (0.25, 0.34)	228	0.40 (0.35, 0.45)	**0.75 (0.61, 0.92)**
Baseball						
Competition	90	1,155,822	0.78† (0.62, 0.94)	72	0.62|| (0.48, 0.77)	1.25 (0.91, 1.73)
Practice	61	2,134,006	0.29‡ (0.21, 0.36)	77	0.36 (0.28, 0.44)	0.79 (0.56, 1.12)
Total	151	3,289,828	0.46§ (0.39, 0.53)	149	0.45¶ (0.38, 0.53)	1.01 (0.80, 1.28)
Softball						
Competition	11	846,153	0.13 (0.05, 0.21)	25	0.30 (0.18, 0.41)	**0.44 (0.20, 0.93)**
Practice	9	1,602,489	0.06 (0.02, 0.09)	54	0.34 (0.25, 0.43)	**0.17 (0.07, 0.34)**
Total	20	2,448,642	0.08 (0.05, 0.12)	79	0.32 (0.25, 0.39)	**0.25 (0.15, 0.42)**

*IRR*, incidence rate ratio (compares pitchers to nonpitchers); *CI*, confidence interval.

Bolded values indicate statistical significance.

∗ Compares pitchers to nonpitchers within same sport.

† IRR = 5.99 (95% CI: 3.19, 12.43) significantly greater for baseball compared to softball *P* < .001.

‡ IRR = 5.09 (95% CI: 2.51, 11.66) significantly greater for baseball compared to softball *P* < .001.

§ IRR = 5.62 (95% CI: 3.51, 9.46) significantly greater for baseball compared to softball *P* < .001.

|| IRR = 2.11 (95% CI: 1.32, 3.47) significantly greater for baseball compared to softball *P* < .001.

¶ IRR = 1.40 (95% CI: 1.06, 1.87) significantly greater for baseball compared to softball *P* < .001.

**Table VI:** Factors associated with elbow injury occurrence.

Overall	Odds ratio (95% CI)	95% CI	*P* value
Position (Nonpitcher reference)	1.75	1.05, 2.93	**.03**
Event (Competition reference)	2.07	1.27, 3.36	**.003**
Age (yr)	0.87	0.72, 1.06	.17
Height (cm)	1.03	0.94, 1.13	.50
Weight (kg)	1.01	1.00, 1.02	.13
Sport (Baseball reference)	0.70	0.38, 1.27	.24

*CI*, confidence interval.

Bolded values indicate statistical significance.

## Discussion

In this study, data from the National High School Sports-Related Injury Surveillance Study was analyzed to provide an updated assessment of elbow and forearm injuries amongst HS baseball and softball players from the 2005-2006 season through the 2018-2019 season, which was the most recent season available at the time of this study. There were a total of 518 injuries reported out of 5,738,470 AEs, giving a combined injury rate of 0.90/10,000 AEs for both baseball and softball. There was a significant trend of increasing injuries in baseball by 0.044/10,000 AE per year the course of the study. There was no significant trend for softball. These findings are similar to prior studies using the same dataset, finding an overall rate of 0.86/10,000 AEs and 0.92/10,000 AEs.^7^^,^^10^ Notably, this is lower than combined elbow and forearm injuries reported using NATION data on the 2011-2012 through 2013-2014 years in softball players, which was 0.84/10,000 AEs.^12^ This is likely attributable to reporting differences between the datasets. NATION uses an electronic medical record to document as opposed to a website based reporting. It is based on a common data element export standard to extract injury data from standard reporting. This may result in higher levels of injury reporting.^2^ Our study also differed from prior literature in detecting a small but significant increase in year-over-year baseball injuries. Although Saper et al detected a nonsignificant increase in elbow rates, the injury rates collected in years after their collection period trended higher.^10^

Baseball was found to have a significantly higher rate of forearm and elbow injury than softball. This pertained to both competition and practice. The softball injury rate of 0.60/10,000 AEs is well within the range of reported in a recent systematic review.^4^ One prior epidemiologic study by Pytiak et al on elbow injuries found similar results with about double the injury rate reported for baseball.^7^ This has been attributed to differences in windmill pitching biomechanics, resulting in less strain and thus lower rates of injuries.^1^ It is well established that baseball pitching induces great valgus stress at the elbow, even in youth athletes.^9^ It is suggested that this stress and possible high pitch counts and/or improper mechanics may result in injuries to baseball pitchers.^5^ There are likely physiologic differences as well, given the gender divide between HS baseball and softball. For providers caring for both athletes, it is important to take account for these nuances.

Pytiak et al also found that there were similar rates of injury in practice and competition for softball but more than twice the rate of injury in baseball competition.^7^ Prior studies have also detected significantly higher rates of injury in competition than practice.^6^^,^^8^ This itself is not that surprising, given the likely increased intensity of play during competition, even in noncontact sports. However, it is less clear why this trend is not found in softball. Our data shows that the difference between practice and competition in baseball was largely driven by pitchers, who had 2.7 times as many injuries in competition than practice, whereas nonpitchers had only 1.7 times the number of injuries. Softball players actually experienced similar relative rates of injuries with 2.3 times the rate of injury in competition vs. 1.6 in practice. However, the aggregate injury rate for softball nonpitchers was nearly 4 times greater than for pitchers leading to our results.

While many injuries resulted in less than a week before return to play for both sports, baseball had significantly higher rates of injury resulting in 10 days or more missed. This difference in severity of injuries has been depicted in other studies, with higher rates of surgery and greater time lost.^7^ This finding may be reflective of the kinds of injuries sustained in baseball players. Our data shows that baseball players have a higher rate of fractures and sprains, which may include significant ligamentous injuries such as rupture of the medial UCL.

This study has several limitations to consider. Only school-sanctioned events were captured, so exposures in travel or other leagues were not included. This data set did not indicate which players did or did not participate in other leagues either. Future studies accounting for additional play would be helpful to truly calculate the increase in exposure and impact on risk. By using an online reporting system, there may be logistical barriers to reporting of all injuries and may underestimate the total number that occurred, especially minor issues that do not result in time away from sport. In addition, there may be discrepancies in injury categorization, particularly in injuries that do not require imaging or intervention. Lastly, there is a selection bias in using schools with ATs who are affiliated with the National Athletic Trainers' association who were willing to participate. However, this may be considered to result in maintained quality standards.^10^ These limitations are common in epidemiological studies based on large datasets.^7^^,^^10^^,^^12^ Future research may benefit from broader reporting systems with more streamlined reporting to capture all injuries and improve external validity. If not an impediment, guidelines for consistency in diagnosis may also be of value.

## Conclusions

Based on data from the National High School Sports-Related Injury Surveillance Study, there is an overall rate of elbow and forearm injuries of 0.90/10,000 AEs in HS baseball and softball athletes. Unlike prior epidemiological studies, this analysis shows that baseball has experienced a significant uptrend in elbow and forearm injuries from 2005 to 2019. Baseball had a higher rate of injury and a higher rate of injuries resulting in 10 or more days missed compared to softball. While there are no significant differences in injury rates between pitchers and nonpitchers across all exposures for baseball, softball nonpitchers had significantly greater injury rates for all injuries than pitchers. To best prevent injuries in these HS athletes, it may be prudent to develop sport- and position-specific interventions.

## Disclaimers

Funding: No funding was obtained for this study.

Conflicts of interest: The authors, their immediate families, and any research foundation with which they are affiliated have not received any financial payments or other benefits from any commercial entity related to the subject of this article.

## References

[1] Barrentine S.W., Fleisig G.S., Whiteside J.A., Escamilla R.F., Andrews J.R. (1998). Biomechanics of windmill softball pitching with implications about injury mechanisms at the shoulder and elbow. J Orthop Sports Phys Ther.

[2] Dompier T.P., Marshall S.W., Kerr Z.Y., Hayden R. (2015). The national athletic treatment, injury and outcomes network (NATION): methods of the surveillance program, 2011-2012 through 2013-2014. J Athl Train.

[3] Drew M.K., Finch C.F. (2016). The relationship between training load and injury, illness and soreness: a systematic and literature review. Sports Med.

[4] Enata N.M., Inclan P.M., Brophy R.H., Knapik D., Smith M.V. (2025). The incidence of shoulder and elbow injuries in high school and collegiate softball athletes: a systematic review. Sports health: a multidisciplinary approach. Sports Health.

[5] Popchak A., Burnett T., Weber N., Boninger M. (2015). Factors related to injury in youth and adolescent baseball pitching, with an eye toward prevention. Am J Phys Med Rehabil.

[6] Powell J.W., Barber-Foss K.D. (1999). Injury patterns in selected high school sports: a review of the 1995-1997 seasons. J Athl Train.

[7] Pytiak A.V., Kraeutler M.J., Currie D.W., McCarty E.C., Comstock R.D. (2018). An epidemiological comparison of elbow injuries among United States high school baseball and softball players, 2005-2006 through 2014-2015. Sports Health.

[8] Rechel J.A., Yard E.E., Comstock R.D. (2008). An epidemiologic comparison of high school sports injuries sustained in practice and competition. J Athl Train.

[9] Sabick M.B., Torry M.R., Lawton R.L., Hawkins R.J. (2004). Valgus torque in youth baseball pitchers: a biomechanical study. J Shoulder Elbow Surg.

[10] Saper M.G., Pierpoint L.A., Liu W., Comstock R.D., Polousky J.D., Andrews J.R. (2018). Epidemiology of shoulder and elbow injuries among United States high school baseball players: school years 2005-2006 through 2014-2015. Am J Sports Med.

[11] Savoie F.H., O’Brien M.J. (2024). Medial elbow injuries in the throwing athlete. J Shoulder Elbow Surg.

[12] Snyder Valier A.R., Bliven K.C.H., Gibson A., Simon J., Dompier T.P., Wasserman E.B. (2020). Non–time-loss and time-loss softball injuries in secondary school athletes: a report from the national athletic treatment, injury and outcomes network (NATION). J Athl Train.

[13] Solomon J, Farrey T (2023). Aspen institute state of play.

[14] Swindell H.W., Trofa D.P., Confino J., Sonnenfeld J.J., Alexander F.J., Ahmad C.S. (2020). Performance in collegiate-level baseball players after elbow ulnar collateral ligament reconstruction. Orthop J Sports Med.

[15] Wasserman E.B., Sauers E.L., Register-Mihalik J.K., Pierpoint L.A., Currie D.W., Knowles S.B. (2019). The first decade of web-based sports injury surveillance: descriptive epidemiology of injuries in US high school boys’ baseball (2005-2006 through 2013-2014) and national collegiate athletic association men’s baseball (2004-2005 through 2013-2014). J Athl Train.

[16] Xanthopoulos M.S., Benton T., Lewis J., Case J.A., Master C.L. (2020). Mental health in the young athlete. Curr Psychiatry Rep.

